# Characteristics of lower respiratory microbiota in children’s refractory *Mycoplasma pneumoniae* pneumonia pre- and post-COVID-19 era

**DOI:** 10.3389/fcimb.2024.1438777

**Published:** 2025-01-21

**Authors:** Zhimin Xi, Jinglong Chen, Libo Wang, Aizhen Lu

**Affiliations:** Division of Pulmonology, Children’s Hospital of Fudan University, Shanghai, China

**Keywords:** refractory *M. pneumoniae* pneumonia (RMPP), COVID-19, children, lower respiratory tract, microbiota

## Abstract

**Introduction:**

Little was known about the characteristics of low respiratory tract (LRT) microbiota of refractory *M. pneumoniae* pneumonia (RMPP) in children before and after the COVID-19 pandemic.

**Methods:**

Forty-two children diagnosed with RMPP in 2019 (Y2019 group) and 33 children diagnosed with RMPP in 2023 (Y2023 group), entered into the study. The characteristics of the clinical findings were examined, and the LRT microbiota was analyzed by metagenomic next generation sequencing.

**Results:**

The ratio of consolidate, atelectasis, lung necrosis, and erythema multiforme in Y2023 group was significantly higher than that in Y2019 (*P*<0.05). *Mycoplasmoides pneumoniae* was the top species of the LRT microbiota in both groups. The rate of macrolide resistance MP in Y2023 was significantly higher than that in Y2019 (*P*<0.05), and the mutant site was all 23S rRNA A2063G. There were no significant differences in α-diversity and β-diversity of LRT microbiota between Y2019 and Y2023 group. *Trichoderma citrinoviride, Canine mastadenovirus A, Ralstonia pickettii, Lactococcus lactis, Pseudomonas aeruginosa* were the biomarkers of LRT microbiota in children with RMPP of Y2023. The abundance of *Mycoplasmoides pneumoniae* positively correlated with the levels of D-dimer and LDH, negatively correlated with the counts of CD3^+^ T cells, CD8^+^ T cells, CD19^+^ B cells and CD16^+^CD56^+^ NK cells.

**Discussion:**

Our study showed that high abundance of MP was correlated with the severity of RMPP and decrease of immune cells. *Trichoderma citrinoviride, Canine mastadenovirus A, Ralstonia pickettii, Lactococcus lactis, Pseudomonas aeruginosa* were the biomarkers in microbiota of LRT in children with RMPP post COVID-19 era.

## Introduction

*M. pneumoniae* (MP) is a significant pathogen of community acquired pneumonia in children (Rogozinski et al., 2017; Leung et al., 2018). Although MP infections are typically self-limited, they can result in extrapulmonary complications or severe pneumonia that needs to be treated in intensive care (Lee et al., 2021). Studies showed that the MP epidemic spikes every 3 to 7 years, most likely as a result of antigenic shift or decreased herd immunity (Lenglet et al., 2012). China’s most recent MP outbreak peaked in 2019 (Wang et al., 2022). Following the COVID-19 pandemic, there was a notable decrease in the number of children with MP infection, owing to the implementation of strong mitigation measures (Wang et al., 2022; Zhang et al., 2022). China has had another outbreak of MP infection since last August 2023 (Meyer Sauteur et al., 2024; Yan et al., 2024). During both epidemics, the rates of refractory *Mycoplasma pneumoniae* pneumonia (RMPP) were very high (Wang et al., 2022; Meyer Sauteur et al., 2024; Yan et al., 2024).

Recently, with advances in nucleic acid extraction techniques and bioinformatics analysis, breakthroughs had been made in the field of metagenomics of respiratory specimens, and the respiratory microbiota has been further studied. Microbe–microbe interactions can significantly impact the etiology, pathogenesis, and frequency of respiratory diseases (Wu and Segal, 2018), and the respiratory microbiota may strengthen the immunity and offer defense against the colonization of pathogens (Esposito and Principi, 2018; Yagi et al., 2021). Several investigations demonstrated how the respiratory microbiota changes following COVID-19 infection and how these changes relate to the prognosis of diseases (Xu et al., 2021; Zhou et al., 2023; Romani et al., 2024). However, little is known about the lower respiratory tract (LRT) microbiota of RMPP in children prior to and following the COVID-19 pandemic. Therefore, this study aimed to investigate the lower respiratory microbiota of RMPP both before and after the COVID-19 pandemic. We believed that through the analysis of microbial composition, we would have the opportunity to understand the complex distribution and interactions of microbiota, and to elucidate the relationship between RMPP infection and the host microenvironment.

## Materials and methods

### Subjects and groups

This is a retrospective study. The RMPP cases who were admitted to the Children’s Hospital of Fudan University both before and after the COVID-19 pandemic were included. The RMPP cases prior to the COVID-19 pandemic who were admitted to hospitals between July 1 and November 30, 2019 were categorized as Y2019. The RMPP cases following the COVID-19 pandemic who were admitted to hospitals between August 1 and December 31, 2023 were categorized as Y2023. RMPP is referred to as clinical manifestations and the pulmonary images of MP pneumonia, showing deterioration after regular macrolide antibiotics treatment for more than 7 days (Chen et al., 2021). All RMPP cases received bronchoscopy, and filled out informed consent form for bronchoscopy was obtained from the guardians. MP infection was confirmed by serological tests, testing positive for MP IgM or an IgG antibody titer ≥1:160 or with a ≥4-fold increase (SeroMPTM IgM and SeroMPTM IgG Test Kit, Savyon Diagnostics Ltd.), and by polymerase chain reaction showing >2,500 copies of *M. pneumoniae* genome per milliliter in the nasopharyngeal aspirate or sputum or bronchoalveolar lavage fluid (BALF) (*M. pneumoniae* nucleic acid amplification fluorescence detection kit, Daan Gene Co., Ltd., Guangzhou) (Chen et al., 2021). Patients who had any other pathogens detected in their blood, nasopharyngeal aspirate, sputum, or BALF by culture, viral antigen detection assays, or serum tests were excluded, as were patients who had heart disease, immunodeficiency, chronic illnesses, or were taking immunosuppressive medications. The ethical application of this study was approved by the ethics committee of the Children’s Hospital of Fudan University on February 28, 2024 (no. 2024-87).

### Clinical characteristics and laboratory findings

Age and gender were gathered, along with information about fever days, respiratory failure, expulmonary manifestation like erythema multiforme, and lung image manifestations like atelectasis, lung necrosis, and consolidate pleural effusion. The laboratory findings before bronchoscopy were also recorded, which comprised of white blood cell (WBC) counts, neutrophil percentage (Neu%), monocyte percentage (Mon%), C-reactive protein (CRP), erythrocyte sedimentation rate (ESR), lactate dehydrogenase (LDH), levels of procalcitonin (PCT), D-dimer, alanine aminotransferase (ALT), lymphocyte subpopulations including CD3^+^ T cells, CD3^+^CD4^+^T cells, CD3^+^CD8^+^T cells, CD16^+^CD56^+^ NK cells, and CD19^+^ B cells.

### BALF specimen collection

Bronchoscopy was performed under conscious intravenous sedation with midazolam. Topical anesthesia of the larynx, trachea, and carina was achieved with 2% lidocaine (Sanchine, China). The bronchoscope was wedged in the lesion’s segment or lobe, and the lavage was performed with three aliquots of sterile saline (Baxter, China), 1 mL/kg each, with a suction pressure of 100 mmHg. All BALF samples were then immediately processed and stored according to the requirements of the laboratory.

### DNA extraction and metagenomic sequencing

DNA extraction from BALF was performed as described (Chen et al., 2021). Briefly, 1 mL BALF was digested by using 50 μL protease K at 60°C for 20 min and then placed at 4°C for 5 min. The sample was transferred into a sterile 5-mL tube, followed by brief centrifugation, and the DNA was extracted using TIANamp Magnetic DNA Kit (DP710-t2, Tiangen, China) according to the manufacturer’s protocol. The quantity and quality of DNA were evaluated using Qubit 2.0 Fluorometers and Nanodrop 8000 Spectrophotometers (Thermo Fisher Scientific, USA), respectively. DNA library construction was performed according to the instructions of Hieff NGS OnePot II DNA Library Prep Kit (Yeasen Biotech, Shanghai, China). The Agilent 2100 Bioanalyzer system (Agilent, Santa Clara, CA, USA) and the Qubit dsDNA HS Assay Kit (Thermo Fisher Scientific Inc., Waltham, MA, USA) were used to control the DNA library fragment sizes and concentrations. Sequencing was performed according to the manufacturer’s manual using the following: first, the library was thermally denatured to form a single-stranded DNA, which was circularized to form a single-stranded circular structure; second, the DNA was amplified using rolling circle amplification technology to form a DNA Nano Ball (DNB, DNA Nano Ball); finally, we completed sequencing in the single-end 50-bp sequencing mode using DIFSEQ-200 (Dinfectome, Nanjing, China). NTCs were also included in the library preparation and sequencing process.

### Bioinformatics

Raw sequencing data was split by bcl2fastq2 (version 2.20), and clean reads were screened using Trimmomatic (version 0.36) by removing low-quality reads, adapter contamination, duplications, and short (length <36 bp) reads (Chen et al., 2021). This study generated a total of 1.32G reads of data from 76 samples, with an average of 17.43M reads per sample, and the Q30% average is 90.15%. Bowtie2 (version 2.2.6) was then used to align with the human genome (hs37d5), and the unaligned sequences were retained. Kraken2 (version 2.0.7) was used to identify the species contained in the sample, and Bracken (version 2.5.0) was used to predict the actual relative abundance of the species in the sample. The microorganism genome database containing genomes or scaffolds of bacteria, fungi, viruses, and parasites was downloaded from GenBank (release 238, ftp://ftp.ncbi.nlm.nih.gov/genomes/genbank/).

### Statistical analysis

The statistical analyses were performed using SPSS software (IBM, version 25.0); *P <*0.05 was defined as statistically significant. Other statistical analyses were performed using R software (v4.0.1), and the parameters were all default. Alpha diversity was measured using Shannon Index, pielou index, Simpson Index, and Chao1 index (“diversity” function in “vegan” R package). Beta diversity in Y2019 and Y2023 groups was analyzed by using the “vegan” R package to perform permutational multivariate analysis of variance (PERMANOVA) and visualized with principal coordinate analysis (PCoA, “pcoa” function in “ape” R package), principal component analysis (PCA, “prcomp” function), and non-metric multidimensional scaling (NMDS, “metaMDS” function in “vegan” R package) plot. Beta diversity was also evaluated using the Bray–Curtis distances, compared by Wilcoxon rank-sum test between the Y2019 and Y2023 groups. Kruskal–Wallis rank-sum test (“kruskal.test” R package) was used to test the differential relative abundance of taxonomic groups at the genus and species levels. The “cor.test” R package was used to assess Spearman’s correlations between clinical characteristics and the relative abundances of species, and FDR correction was used to adjust all *P* values. The key taxa difference between the Y2019 and Y2023 groups was determined by using the linear discriminant analysis (LDA) of effect size (LEfSe, “LEfSe” R package) and RandomForest (“RandomForest” R package) analysis.

## Results

### Clinical characteristics

In this study, 42 participants, comprising 17 male participants and 25 female participants, were assigned to the Y2019 group, while 33 participants, comprising 16 male participants and 17 female participants, were assigned to the Y2023 group. There was no significant difference in gender distribution. The age of the Y2023 group was older than that of the Y2019 group (79.12 ± 30.03 months vs. 60.88 ± 32.12 months, *P* < 0.05) (**Table 1**). There was no significant difference in fever days between the two groups (*P* > 0.05) (**Table 1**). The ratio of consolidate, atelectasis, lung necrosis, and extrapulmonary manifestation such as erythema multiforme in the Y2023 group was significantly higher than that in Y2019 (*P* < 0.05) (**Table 1**).

**Table 1:** Clinical characteristics.

Variable	Y2019 (*n* = 42)	Y2023 (*n* = 33)	*P* value
Male/female	17/25	16/17	0.48
Age (months)	60.88 ± 32.12	79.12 ± 30.03	0.01
Fever days	9.90 ± 5.51	10.39 ± 7.35	0.11
Consolidate	17/42	27/33	0.00
Atelectasis	10/42	25/33	0.00
Pleural effusion	10/42	12/33	0.23
Lung necrosis	2/42	7/33	0.03
Respiratory failure	4/42	1/33	0.23
Erythema multiforme	0/42	6/33	0.01

### Laboratory findings

There were no significant differences in WBC count, NEU%, Mon%, or levels of CRP, ESR, PCT, D-dimer, and ALT between the two groups (*P* > 0.05) (**Table 2**). The level of LDH in Y2019 was higher than that in the Y2023 group (484.31 ± 248.28 vs. 353.86 ± 118.53, *P* < 0.05) (**Table 2**). In cellular immunity, there were no significant differences in the counts of CD3^+^ T cells, CD3^+^CD4^+^ T cells, CD3^+^CD8^+^ T cells, CD4^+^ T cells/CD8^+^ T cells, and CD19^+^ B cells between the two groups (*P* > 0.05) (**Table 2**). The CD16^+^CD56^+^NK cells in the Y2019 group were significantly higher than that in the Y2023 group (*P* < 0.05) (**Table 2**).

**Table 2:** Laboratory findings.

Variable	Y2019 (*n* = 42)	Y2023 (*n* = 33)	*P* value
WBC counts (×10^9^/L)	12.74 ± 4.72	11.14 ± 5.20	0.16
NEU (%)	66.68 ± 17.64	63.18 ± 18.41	0.38
Mon (%)	17.59 ± 9.01	25.28 ± 18.83	0.19
CRP	34.06 ± 35.44	20.72 ± 26.58	0.07
ESR	53.61 ± 30.56	53.70 ± 32.14	0.99
PCT	0.30 ± 0.46	0.14 ± 0.30	0.10
LDH (IU/mL)	484.31 ± 248.28	353.86 ± 118.53	0.01
D-dimer (mg/L)	2.94 ± 4.40	2.40 ± 3.95	0.57
ALT (U/L)	30.17 ± 26.76	26.36 ± 17.50	0.48
Cellular immunity			
CD3^+^ T cells	1,668.64 ± 1,073.69	1,586.52 ± 1,570.40	0.80
CD3^+^CD4^+^ T cells	1,002.97 ± 734.63	974.48 ± 1,263.76	0.91
CD3^+^CD8^+^ T cells	588.61 ± 345.50	562.52 ± 401.64	0.77
CD4^+^/CD8^+^ T cells	1.71 ± 0.71	1.60 ± 0.89	0.56
CD16^+^CD56^+^ NK cells	250.37 ± 206.97	153.58 ± 105.26	0.02
CD19^+^ B cells	840.96 ± 670.63	575.21 ± 659.33	0.10
Macrolide resistance	1/42	9/33	0.00

### Analysis of LRT microbiota in children with RMPP

#### LRT microbiota composition

**Figure 1:**
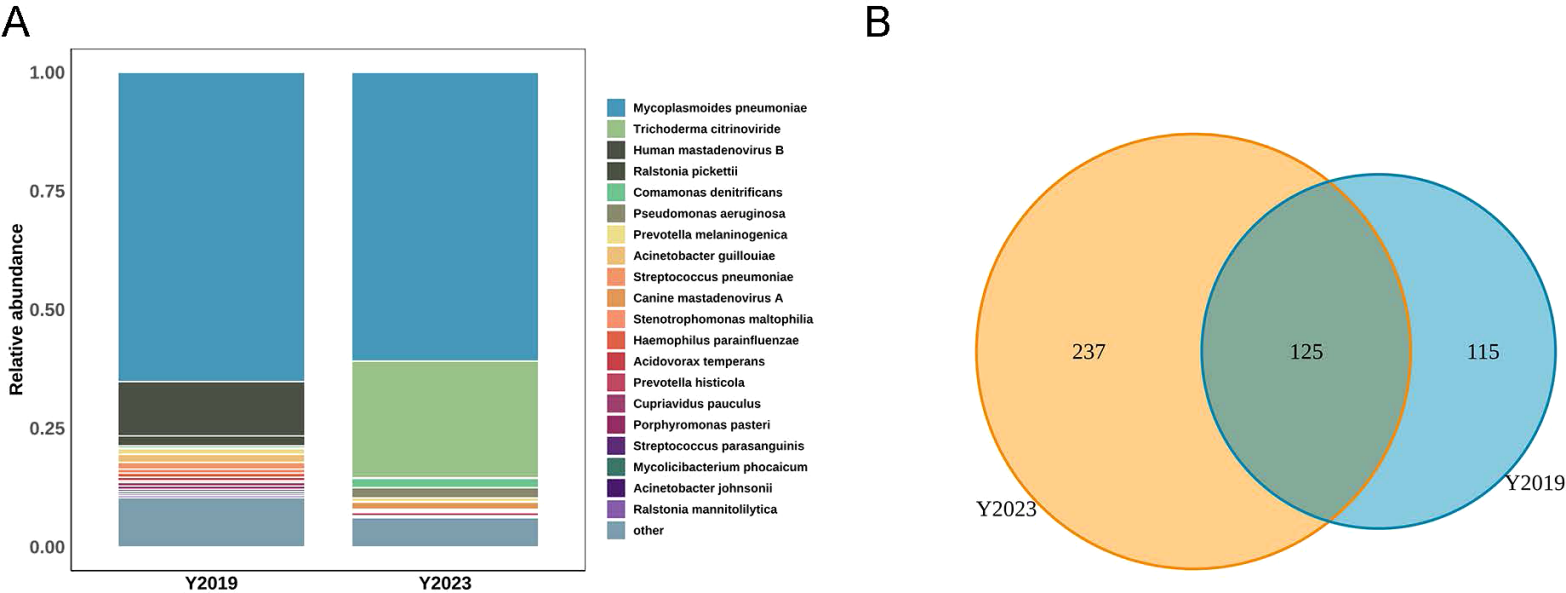
LRT microbiota composition in children with RMPP. **(A)** Relative abundance of species between Y2019 group and Y2023 group, and *Mycoplasmoides pneumoniae* was the top species in both groups. **(B)** Venn diagram displaying the overlaps between groups showed that 125 of the total number of 477 species were shared in both groups, while 237 were unique for the Y2023 group. LRT, lower respiratory tract; RMPP, refractory *Mycoplasma pneumoniae* pneumonia.

We displayed the top 20 most abundant species between the two study groups. *Mycoplasmoides pneumoniae* was the top species of the LRT microbiota in both groups, accounting for 65.16% of the total sequences in the Y2019 group and accounting for 60.83% of the total sequences in the Y2023 group (**Figure 1A**). By binding to the 23S rRNA and preventing protein synthesis, macrolides inhibited MP development. The next-generation sequencing data showed that the mutant site was all 23S rRNA A2063G, and the rate of macrolide resistance MP in Y2023 (9/33) was significantly greater than that in Y2019 (1/42) (*P* < 0.05) (**Table 2**). Human mastadenovirus B ranked as the second top species in the Y2019 group, accounting for 11.42% of the total sequences. *Trichoderma citrinoviride* ranked as the second top species in the Y2023 group, accounting for 24.57% of the total sequences. A Venn diagram illustrates that there were 477 species in total, with 125 species shared by the two groups. Notably, 115 species were exclusive to Y2019, while 237 species were exclusive to Y2023 (**Figure 1B**).

#### α-diversity and β-diversity of LRT microbiota

**Figure 2:**
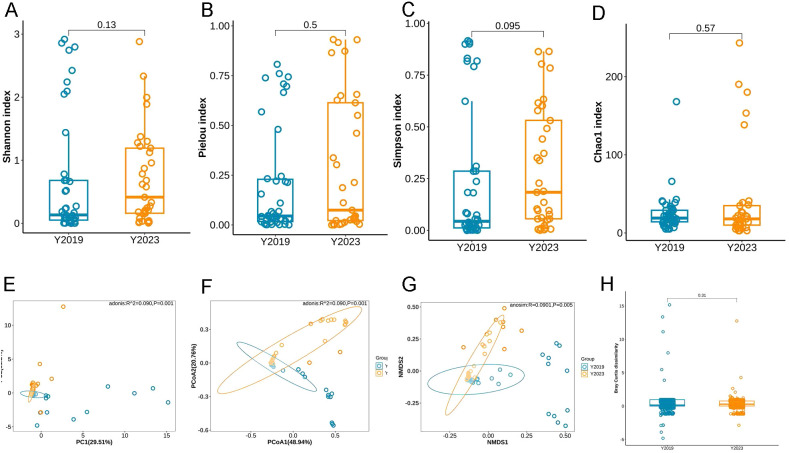
α-diversity and β-diversity of LRT microbiota in children with RMPP. α-diversity was estimated by using the **(A)** Shannon index, **(B)** pielou index, **(C)** Simpson index, and **(D)** Chao1 index, and there was no significant difference between the two groups. β-diversity was estimated by using **(E)** PCA, **(F)** PCoA, **(G)** NMDS, and **(H)** Bray–Curtis distance, and there was no significant difference between the two groups. LRT, lower respiratory tract; RMPP, refractory mycoplasma pneumoniae pneumonia; PCA, principal component analysis; PCoA, principal coordinate analysis; NMDS, non-metric multidimensional scaling.

α-diversity was calculated with Shannon index, Pielou index, Simpson index, and Chao1 index, which stand for the microbiota’s richness and evenness. There were no significant differences in Shannon index, Pielou index, Simpson index, or Chao1 index between the Y2019 and Y2023 groups (**Figures 2A–D**), suggesting that there was no significant microbial diversity between the two groups. An overview of the BALF microbiome was provided by the examination of its β-diversity using principal component examination (PCA), principal coordinate analysis (PCoA), and non-metric multidimensional scaling (NMDS) (**Figures 2E–G**). The result indicated that there was no significant intragroup difference between the Y2019 and Y2023 groups. This result was confirmed with Bray–Curtis distance (*P* = 0.31, PERMANOVA) (**Figure 2H**).

#### The taxa of LRT microbiota

**Figure 3:**
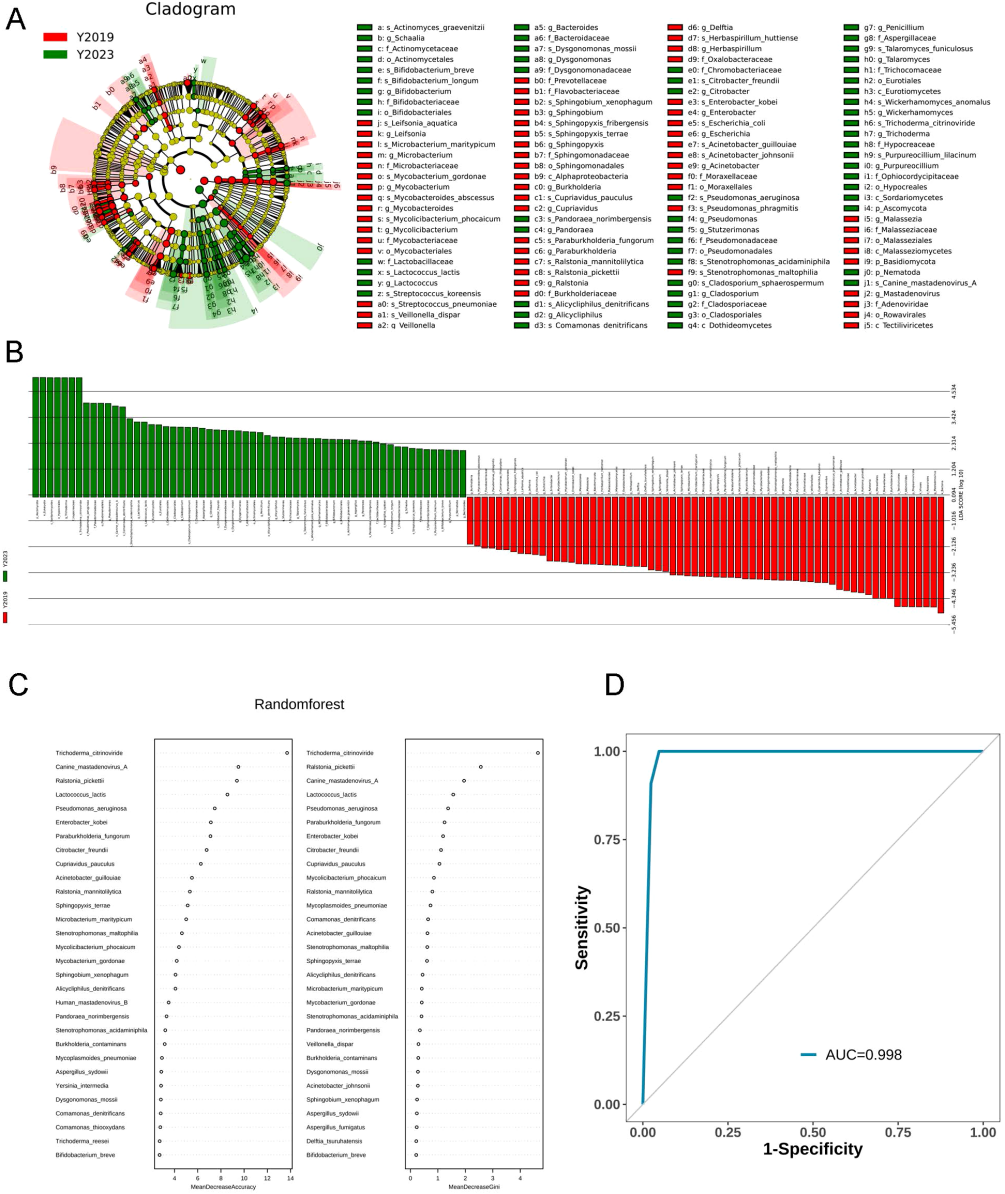
Taxa of LRT microbiota in children with RMPP. LEfSe cladogram and plot representation of taxa differences obtained for the LRT microbiota of the Y2019 and Y2023 groups. Nodes **(A)** and bars **(B)** highlighted in red and green were the significantly more abundant taxa in Y2019 and Y2023. A random forest classifier model between the two groups was constructed to assess the potential of microbial biomarkers. *Trichoderma citrinoviride*, *Canine mastadenovirus A*, *Ralstonia pickettii*, *Lactococcus lactis*, and *Pseudomonas aeruginosa* were the top five species to distinguish Y2023 from Y2019 group **(C)**, with an AUC of 0.998 by ROC analysis **(D)**. LRT, lower respiratory tract; RMPP, refractory mycoplasma pneumoniae pneumonia; LEfSe, Linear discriminant analysis effect size; LDA, linear discriminant analysis.

The top 20 most abundant species between the two study groups were compared. Both groups were enriched with *Mycoplasmoides pneumoniae*. Linear discriminant analysis effect size (LEfSe) was performed to elucidate the maximum different microbes between the two groups (**Figure 3A**). The results were ranked by using linear discriminant analysis (LDA) score histogram (**Figure 3B**). This analysis revealed that *Trichoderma citrinoviride*, *Pseudomonas aeruginosa*, *Canine mastadenovirus A*, and *Comamonas denitrificans* in the Y2023 group significantly contributed to microbiota differences compared with that in the Y2019 group. However, in the Y2019 group, *Ralstonia mannitolilytica*, *Acinetobacter guillouiae*, *Streptococcus pneumoniae*, *Cupriavidus pauculus*, *Stenotrophomonas maltophilia*, and *Mycolicibacterium phocaicum* significantly contributed to microbiota differences compared with that in the Y2023 group. The potential of the microbial biomarkers was then evaluated by building a random forest classifier model between the two groups. Five species were selected as the optimal biomarker set. The result revealed that the top five species to distinguish Y2023 group from Y2019 group were *Trichoderma citrinoviride*, *Canine mastadenovirus A*, *Ralstonia pickettii*, *Lactococcus lactis*, and *Pseudomonas aeruginosa* (**Figure 3C**). With an AUC of 0.998 by ROC analysis (**Figure 3D**), the random forest classifier model with all species demonstrated a strong diagnostic potential in differentiating Y2023 from Y2019.

#### Correlation between microbiota and clinical measures

**Figure 4:**
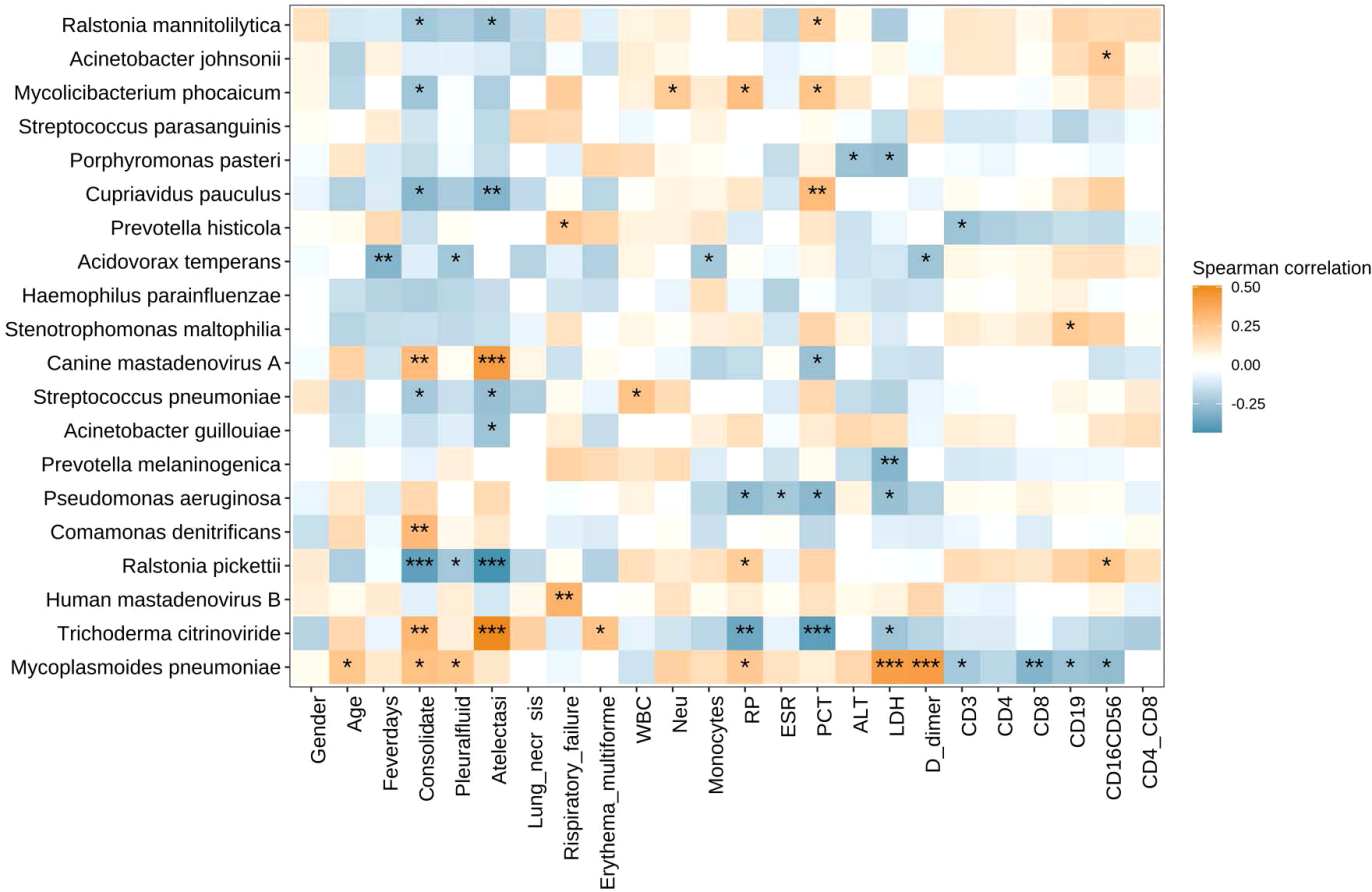
Heatmap of Spearman correlations between clinical measures and microbiota species. The abundance of *Mycoplasmoides pneumoniae* positively correlated with the levels of D-dimer and LDH and negatively correlated with the counts of CD3+ T cells, CD8+ T cells, CD19+ B cells, and CD16+CD56+ NK cells. *P < 0.05, **P < 0.01, and ***P < 0.001. LDH, lactate dehydrogenase.

We also analyzed the correlation between the abundances of respiratory microbiota constituents at the species level and the clinical indices including gender, age, fever days, consolidate, pleural effusion, atelectasis, lung necrosis, respiratory failure, erythema multiforme, and lab indices including WBC, Neu%, Mon%, CRP, ESR, LDH, PCT, D-dimer, ALT, counts of CD3^+^ T cells, CD3^+^CD4^+^T cells CD3^+^CD8^+^T cells, CD16^+^CD56^+^ NK cells, and CD19^+^ B cells, and ratio of CD4+/CD8+ T cells (**Figure 4**). The abundance of *Mycoplasmoides pneumoniae* positively correlated with the levels of D-dimer and LDH and negatively correlated with the counts of CD3^+^ T cells, CD8^+^ T cells, CD19^+^ B cells, and CD16^+^CD56^+^ NK cells.

## Discussion

MP was a common cause of respiratory tract infections, with an epidemical interval of 1–3 years. Prior to the COVID-19 pandemic, the most recent MP epidemics happened in 2019. There has been a global MP pneumonia outbreak since the summer of 2023 (Larcher et al., 2024; Meyer Sauteur et al., 2024; Nordholm et al., 2024; Yan et al., 2024). A direct biological impact of COVID-19 on MP and transient herd immunity are the two most concerning possibilities for a re-outbreak of MP infection (Meyer Sauteur et al., 2022; Meyer Sauteur et al., 2023; Larcher et al., 2024; Meyer Sauteur et al., 2024). In the present study, BALF samples were collected to explore whether the difference of LRT microbiota of children with MPP before and after COVID-19 pandemic contributed to the outbreak of MP in 2023.

In line with previous research, we compared the clinical characteristics of RMPP before and after COVID-19 pandemic and found that the ratio of consolidate, atelectasis, lung necrosis, and extrapulmonary manifestation in the Y2023 group was higher than that in Y2019 (Yan et al., 2024). We also found that the rate of macrolide resistance MP in Y2023 increased significantly. However, the ratio was lower than those of other studies (Jiang et al., 2023; Yan et al., 2024), which may be associated with the different detection techniques.

This study demonstrated that *Mycoplasmoides pneumoniae* was dominant in the microbiota of LRT in children with RMPP before and after the COVID-19 pandemic, exceeding 60% of the entire population in both groups. This result was consistent with our previous study (Chen et al., 2021), and this may be elucidated by the theory that MP could eliminate other bacteria by directly competing for nutrients (Yang et al., 2004; Jiang et al., 2023). Additionally, we found that the abundance of *Mycoplasmoides pneumoniae* positively correlated with both D-dimer and LDH. Since elevated D-dimer and LDH indicate an excessive inflammatory response (Lu et al., 2015; Huang et al., 2021), it was hypothesized that the abundance of *Mycoplasmoides pneumoniae* was positively correlated with the severity of the disease (Chen et al., 2021). In addition, we also found that the abundance of *Mycoplasmoides pneumoniae* was negatively correlated with the counts of CD3^+^ T cells, CD8^+^ T cells, CD19^+^ B cells, and CD16^+^CD56^+^ NK cells. Peng Li et al. also found that peripheral lymphocytopenia existed in severe MPP in children (Peng et al., 2021). This condition was also found in severe respiratory viral infections, such as COVID-19 and influenza (Li et al., 2020). The mechanism underlying lymphopenia after a severe infection remains unknown. In addition to immune activation-induced cell deaths (Lee et al., 2021; Zhang et al., 2022; Zhu et al., 2023), it is reasonable to hypothesize from this result that a high load of *Mycoplasmoides pneumoniae* could directly infect lymphocytes, initiating the cell death of lymphocytes. Of course, further research is required to support the hypothesis.

The lung microbiome is likely to not only impact susceptibility or causes of diseases but also be influenced by disease activities or responses to treatment. The respiratory tract microbiota is a key factor which regulates and shapes pulmonary immune responses (Yagi et al., 2021). Previous studies have shown that children are more vulnerable to acute respiratory tract infections when their respiratory tract microbiome is altered (Langevin et al., 2017; Dubourg et al., 2019; Man et al., 2019). Research also revealed that, even after reaching a stable phase, the microbiome is more susceptible to respiratory infections during childhood and is less resilient (Man et al., 2017; Derrien et al., 2019). In particular, children may experience both immediate and long-term health problems as a result of the persistent dysbiosis of their microbiomes caused by COVID-19 (Man et al., 2017; Derrien et al., 2019). Although there was no significant difference in α-diversity or β-diversity of microbiota between pre- and post-COVID-19 pandemic, we found that the abundance of species other than *Mycoplasmoides pneumoniae* in the microbiota of LRT was very different. *Trichoderma citrinoviride*, *Pseudomonas aeruginosa*, *Canine mastadenovirus A*, and *Comamonas denitrificans* were enriched in the microbiota of LRT post-COVID-19 era. A subsequent investigation revealed that *Trichoderma citrinoviride*, *Canine mastadenovirus A*, *Ralstonia pickettii*, *Lactococcus lactis*, and *Pseudomonas aeruginosa* were the biomarkers in the microbiota of LRT post-COVID-19 era. Among them, *Pseudomonas* has been paid attention to. It was reported that the microbiome of the upper respiratory tract in children with COVID-19 was characterized by *Pseudomonas*-dominated community types, and the dysbiosis of microbiome persists for a very long time (Xu et al., 2021). Another study showed that the microbiota biomarkers of the upper respiratory tract in children with COVID-19 also included *Pseudomonas* (Romani et al., 2024). According to a systematic review, the prevalence of *Pseudomonas aeruginosa* was on the rise before the COVID-19 pandemic and slightly increased during it. Additionally, our study’s data demonstrated that *Pseudomonas* was a biomarker of microbiota in the lung of RMPP children in post-COVID-19 era. All of these data suggested that *Pseudomonas* may be a biomarker of LRT microbiota after the COVID-19 pandemic, which needs further research to validate.

There were limitations of our study. BALF samples were collected for the present study. Few studies have attempted to explore the features of pediatric lower airways before and after COVID-19 infection because of the practical difficulties involved in obtaining LRT samples. BALF needs an invasive process, which has limited its usage in routine respiratory microbiota studies despite the fact that it is less impacted by contamination than sputum. The BALF microbiota data were only examined for patients who had bronchoscopies performed as part of their clinical care. Consequently, no information regarding healthy controls was available. Other limitations were that this study was a single-center research and the sample size was limited. Thus, the results were limited, and large-cohort studies of multicenters should be further designed.

## Conclusions

Our study characterized the features of the LRT microbiota of children with RMPP pre- and post-COVID-19 era and uncovered its association with disease severity. Our results suggested that the high abundance of MP in the LRT was associated with the severity of RMPP and decrease of immune cells. There were differences in the LRT microbiota of children with RMPP pre- and post-COVID-19 era. *Trichoderma citrinoviride*, *Canine mastadenovirus A*, *Ralstonia pickettii*, *Lactococcus lactis*, and *Pseudomonas aeruginosa* were the biomarkers in the microbiota of LRT post-COVID-19 era. This result suggested that a prolonged unstable state of the LRT microbiota post-COVID-19 era may alter the microbiomes of LRT in children and disturb their development.

## Data Availability

The datasets presented in this study can be found at: https://figshare.com/articles/figure/Characteristics_of_low_respiratory_Microbiota_in_Children_s_refractory_Mycoplasma_pneumoniae_pneumonia_pre_and_post_COVID_19_era/28229150.
